# Association between hip and trunk muscle strength and modified single-leg horizontal jump test performance in elite team handball players

**DOI:** 10.3389/fspor.2026.1733358

**Published:** 2026-02-04

**Authors:** Primož Pori, Nejc Šarabon, Marko Šibila, Darjan Spudić

**Affiliations:** 1 Faculty of Sport, University of Ljubljana, Ljubljana, Slovenia; 2 Faculty of Health Sciences, University of Primorska, Izola, Slovenia; 3 Ludwig Boltzmann Institute for Rehabilitation Research, St. Pölten, Austria

**Keywords:** assessment, core, jumping performance, power, prevention, rate of force development, reliability, validity

## Abstract

**Introduction:**

Assessing unilateral jumping performance and its determinants is important in handball, as it underpins key offensive and defensive actions. This study aimed to evaluate the intra-session reliability of a modified single-leg horizontal jump (SLHJ) test in elite players and its associations with hip and trunk strength.

**Methods:**

Eighty elite handball players performed three single-leg horizontal jumps (SLHJs) using both their preferred and non-preferred push-off legs. Jump distance, side deviation, and resultant jump angle were measured as dependent variables. Unilateral maximal and explosive isometric strength of the hip abductors, adductors, extensors, flexors, and lateral trunk muscles were assessed as independent variables.

**Results:**

SLHJ reliability was poor-to-moderate for side deviation and jump angle (ICC_2.k_ = 0.33–0.70) and good-to-excellent for jump distance (ICC_2.k_ = 0.87–0.93). Moderate correlations were observed between non-preferred leg SLHJ distance and explosive hip flexion (*r* = 0.31), and between SLHJ side deviation and hip extension maximal strength (*r* = 0.31). Multiple regression analysis showed that, for the preferred leg, adduction maximal strength predicted distance (*R*^2^ = 6%), and for the non-preferred leg, explosive hip flexion predicted distance (*R*^2^ = 10%), extension and abduction maximal strength predicted side deviation (*R*^2^ = 14.5%), and extension maximal strength predicted jump angle (*R*^2^ = 8.4%).

**Discussion:**

Due to lower reliability, directional SLHJ measures should be interpreted with caution. Hip strength was partially supported as a determinant of SLHJ performance in elite handball players. The modified SLHJ better reflects performance differences related to hip strength in the non-preferred leg.

## 1 Introduction

Team handball is a dynamic, high-intensity intermittent sport characterized by frequent, repeated actions such as jumps, throws, sprints, and changes of direction, often executed under direct physical contact with opponents (1). Modern handball has evolved toward faster offensive and defensive transitions, shorter attack durations, and higher game intensity, increasing the frequency of explosive unilateral actions and placing greater demands on neuromuscular control (2, 3). In addition to the overhead throw, which is the most characteristic unilateral movement pattern in the game, players also perform numerous lower-limb actions, including single-leg jumps, landings, and cutting maneuvers, which are typically performed under physically demanding and unpredictable conditions (4). Such playing demands require optimal neuromuscular control of the lumbopelvic-hip complex, particularly in the frontal and transverse planes. The ability to generate horizontal propulsion and maintain postural stability during unilateral tasks is therefore vital for technical efficiency, peak performance, injury prevention and also return-to-play decisions (5, 6).

The single-leg horizontal jump for distance (SLHJ) is a valid and widely used field-based test to assess unilateral lower limb power, neuromuscular control and functional symmetry or asymmetry in both healthy and injured population (7, 8). The test reflects athletes' ability to generate horizontal force during a single push-off and depends on the contribution of the leg, hip and trunk muscle strength (9, 10). Leg and trunk muscle strength are also key determinants of sprint acceleration, maximal sprinting speed (11), and change-of-direction performance (12). These movement actions are crucial for modern handball player performance due to the fast-paced, multidirectional nature of the game and the frequent need for rapid transitions between offense and defense (2, 13, 14). Although SLHJ distance demonstrates good to excellent test–retest reliability among college students [ICC = 0.87–0.98; (7)], its reliability in handball players remains unknown.

Compared to vertical jump, the SLHJ places greater emphasis on hip and ankle contributions during the propulsive phase (5). This makes it especially relevant for evaluating movement patterns in handball, where jumps often occur unilaterally and in forward directions under unstable and reactive conditions, requiring higher trunk and hip stability to maintain the desired jump trajectory (5). Furthermore, in modern handball, jump shots, sprints, and changes of direction often occur under time-limiting conditions, requiring rapid force production. Therefore, explosive strength, particularly rate of force development (RFD), is a key determinant of jumping performance (15). Assessing RFD through jump tests provides insight into an athlete's ability to translate strength into sport-specific actions and informs training for power and injury prevention. However, the contribution of hip muscle RFD to SLHJ performance in handball players has not been explored.

While jump distance remains the most commonly used variable, it may not fully reflect movement quality or neuromuscular control. Side deviations from the intended jump trajectory, particularly under high-demand conditions, have emerged as sensitive indicators of segmental misalignment, pelvic instability, or compensatory strategies (6, 16). Hip and trunk muscles, particularly hip abductors/adductors and lateral trunk flexors, play a key role in maintaining frontal-plane stability and controlling movement direction during single-leg tasks (17, 18). Weakness or poor control in these regions has been associated with dynamic knee valgus and increased injury risk (19, 20). Lateral trunk flexors such as the quadratus lumborum and external obliques contribute to angular control and segmental alignment during propulsion and landing, acting as a kinetic link for force transfer between the lower limbs (21). Incorporating directional variables such as side deviations from the expected jump trajectory into the SLHJ may provide additional insight into neuromuscular control beyond jump distance alone. Due to the unilateral nature of handball, in which jump shots are predominantly performed using the same leg, single-leg horizontal jump (SLHJ) performance and its relationship with hip and trunk strength may differ between limbs. Therefore, assessing single-leg jump performance separately for the preferred and non-preferred legs is important for identifying strength imbalances, optimizing performance, and reducing injury risk, as observed differences may be a consequence of years of repetitive, sport-specific training.

The aims of this study were to assess the intra-session reliability of the modified SLHJ test in elite handball players and to examine the associations between SLHJ performance variables and hip isometric maximal and explosive strength, as well as maximal lateral trunk strength, for both the preferred and non-preferred legs. In addition to jump distance, side deviation from the expected jump trajectory and, consequently, the resultant jump angle (taking into account both jump distance and side deviation) were evaluated. Given that hip and trunk muscle groups are thought to play a critical role in pelvic stability during single-leg push-off, as well as in generating horizontal propulsion, they are likely important contributors to horizontal jump performance. We hypothesized that SLHJ outcomes would demonstrate good to excellent reliability and that moderate correlations would exist between SLHJ outcomes and hip and lateral trunk strength.

## 2 Materials and methods

### 2.1 Experimental design and participants

This was a cross-sectional study conducted in a single measurement session, with the total duration of approximately 45 min. Participants performed maximal voluntary isometric contractions (MVICs) of the hip and lateral trunk muscles, explosive isometric contractions of the hip muscles, and single-leg horizontal jumps for distance with both limbs. All tests were performed in a randomized order. Prior to testing, participants completed a standardized warm-up protocol supervised by a qualified member of the research team. The warm-up consisted of 10 min of jogging, followed by mobility exercises for the arms, hips, knees, and ankles (10 repetitions each). Participants then performed dynamic stretching exercises targeting the hip flexors, knee extensors, knee flexors, and ankle plantar flexors (10 repetitions each). The warm-up concluded with resistance exercises, including heel raises, squats, and crunches (10 repetitions each). All tests were conducted in a laboratory setting with a parquet floor under controlled environmental conditions (ambient temperature: 21 °C; relative humidity 30%–35%) and were performed by two sports scientists (PhDs) with more than five years of experience in neuromuscular testing.

An *a priori* statistical power analysis was conducted to determine the minimum sample size required for the study using G*Power software (version 3.1; Faul et al., Universität Kiel, Germany). The analysis was based on findings reported by Kollock et al. (22), who observed an average coefficient of determination of *r*^2^ = 0.18 (corresponding to *r* = 0.42) between hip abduction, adduction, flexion, and extension strength and single-leg horizontal jump distance in recreationally active men. Assuming a two-tailed correlation analysis with an alpha level of.05 and a statistical power of 0.80 (*β* = 0.20), the analysis indicated that a minimum of 43 participants was required to reliably detect correlations of comparable magnitude in the present study. To be conservative and to account for the larger number of correlations examined, we ultimately included 80 elite male handball players from the first national Slovenian league, each with at least 10 years of training experience. Their mean (standard deviation) age, height, and body mass were 21.8 (3.9) years, 1.90 (0.06) m, and 92.0 (9.6) kg, respectively. All players were actively engaged in regular handball training, practicing at least five times per week with their clubs over the previous five years, including both pre-season and competitive periods, with approximately 30 official matches (±3) per season. Approximately 70% of the participants also performed regular resistance or preventive training sessions twice per week during this period. The inclusion criteria required participants to be free from musculoskeletal injuries or pain syndromes for at least one week prior to the measurement session, as well as free from any medical conditions that could be aggravated by the testing procedures. In agreement with the coaches, participants were instructed to avoid strenuous activity for two days prior to testing. No dietary restrictions were required. Before data collection, they were fully informed about the study protocol and signed an informed consent form. For underage participants, their parents or legal guardians signed the consent on their behalf. They wore only tight-fitting shorts (mid-thigh length), low-ankle socks, and low-ankle training shoes of their choice to minimize any influence on the testing process. Since single-leg horizontal jumps were routinely performed as part of their physical preparation in regular training, no additional familiarization session was necessary. Testing was conducted during the pre-season period.

### 2.2 Testing procedures

#### 2.2.1 Single-leg horizontal jump for distance test procedure

All participants performed a SLHJ on a parquet surface. Before performing the jump, participants were instructed to execute the take-off as quickly as possible (rapid descent and transition into a horizontal push-off) and to jump as far as possible, landing on the same leg and maintaining the position for an additional two seconds. They were not instructed to jump in a straight line. The non-tested leg was kept slightly flexed at the knee and was not allowed to touch the tested leg. Swinging the non-tested leg during the jump was not permitted. Arm swing, however, was not restricted. Three repetitions were performed with both the preferred and non-preferred legs, preceded by one practice attempt to minimize potential learning effects. The preferred leg was defined as the push-off leg during a jump shot in handball, which is contralateral to the throwing arm. A rest interval of 60 s was provided between individual jumps, alternating between preferred and non-preferred leg.

To prevent participants from targeting a straight-line during landing, jumps were performed in a standardized rectangular area without any markings on the landing surface. A rectangle measuring 3.2 m by 1.7 m was outlined on the floor using adhesive tape 0.05 m thick. Participants stood at the centre of the shorter edge of the rectangle, facing its midpoint, with the front part of their shoes positioned just behind the starting line. The midpoint of each shoe's heel was lightly marked with a pen and aligned with the rectangle's centreline, which extended perpendicularly from the shorter edge.

**Figure 1:**
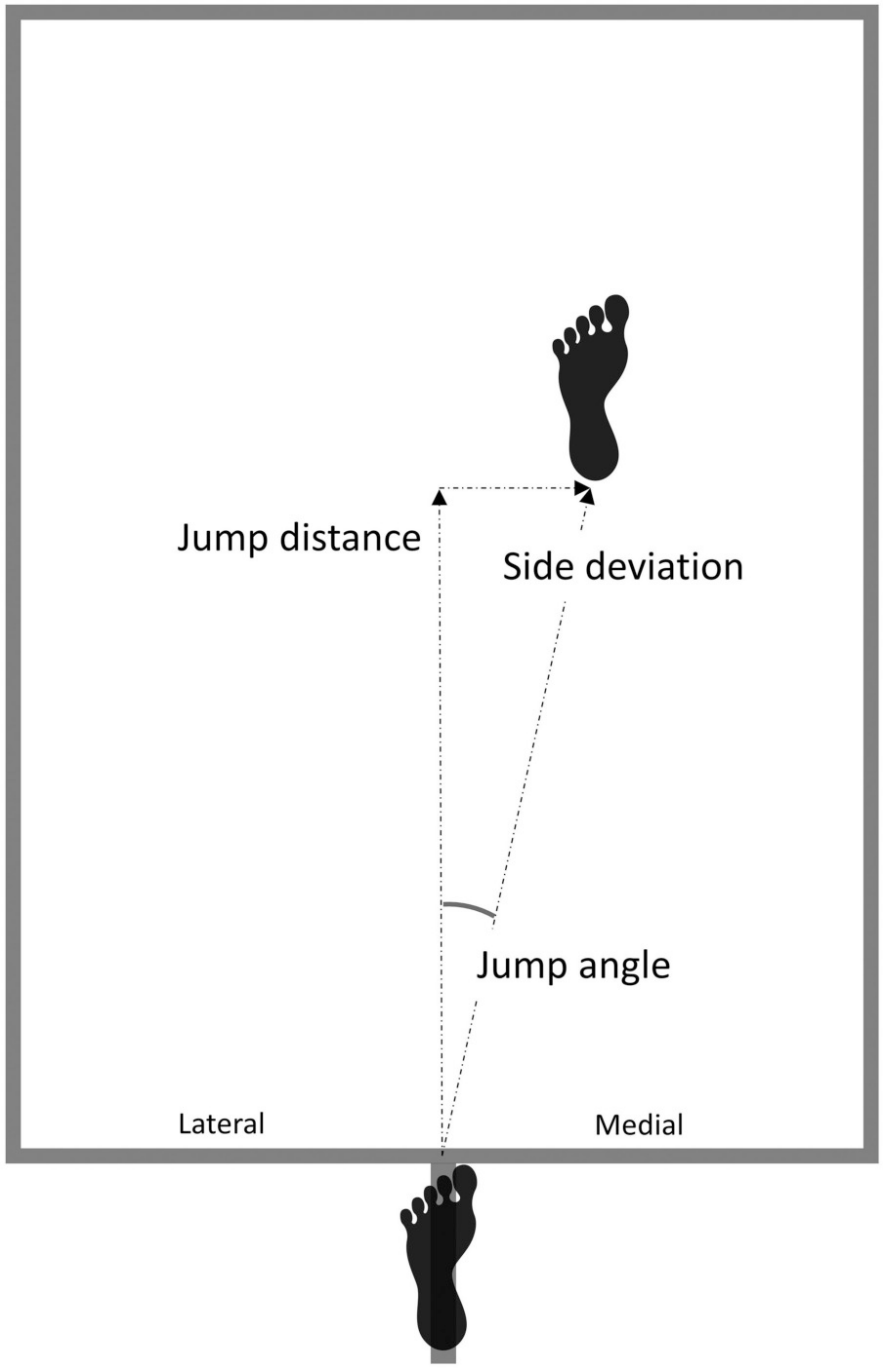
Diagram of the single-leg horizontal jump for distance test setup.

The distance of each jump in metres was measured by two researchers using a steel measuring tape, from the starting line to the pen-marked point on the heel, along a line perpendicular to the starting edge (*Jump distance*). Jump *side deviation* (defined as the sideways displacement from the centreline of the rectangle which can be lateral or medial to the push-off leg) was measured as the perpendicular distance from the longer edge of the rectangle to the pen-marked line at the centre of the heel. The *jump deviation angle* was then calculated as the arctangent of the ratio between the side deviation and the jump distance (both in metres). Negative values for *side deviation* and *jump angle* indicated lateral (outward) side deviation, whereas positive values indicated medial (inward) side deviation in regards to the push-off leg. The testing setup is illustrated in Figure 1. Jump distance and jump side deviation were additionally normalized to participants' leg length to control for anthropometric differences between them. Leg length results were obtained prior to isometric hip strength testing described in the next paragraph. The average of the three repetitions results was used for further analysis for each variable (jump distance, side deviation, and jump angle).

#### 2.2.2 Isometric hip strength and explosive strength assessment

**Figure 2:**
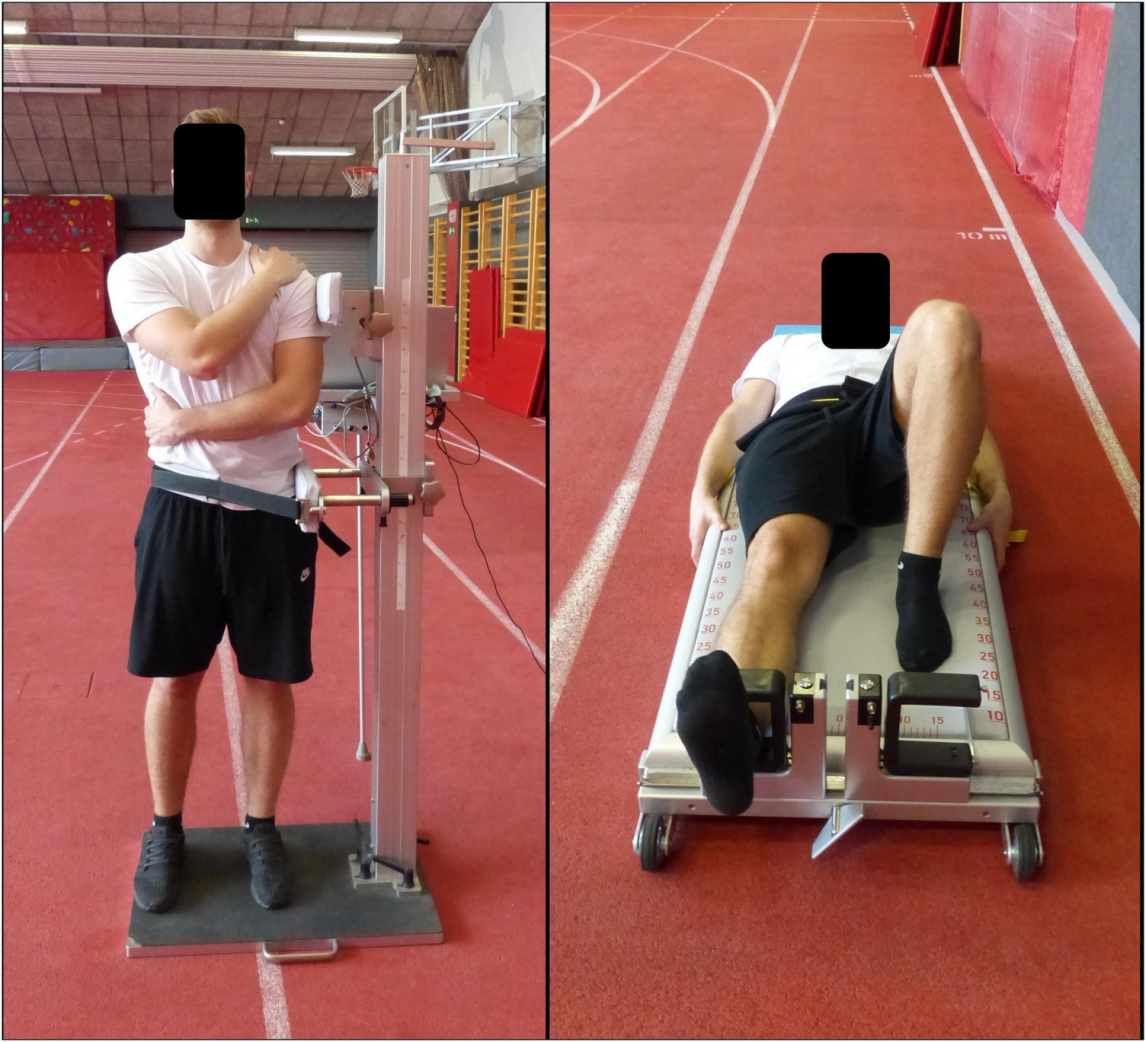
Positioning of participants for trunk lateral flexion strength (left side) and hip strength (right side) measurements.

All hip strength assessments were conducted unilaterally using isometric dynamometer (Muscle Board, S2P d.o.o, Ljubljana, Slovenija), which utilizes U-shaped braces with single-point load cells. The procedure was assessed in terms of intra- and inter-day reliability, and most of the outcomes showed good to excellent reliability scores (23). The braces may be rotated to accommodate the desired task. Hip flexion, adduction, and abduction maximal and explosive strength were measured in the supine position, while hip extension strength and explosive strength was assessed in the prone position, with the hip, knee, and ankle maintained in a neutral position. Testing positions were individually adjusted based on participants' anthropometric characteristics. During all tests, ankles and knees were aligned hip-width apart. Leg lever arm was measured prior each test to the nearest 0.5 cm by measuring the distance from the greater trochanter to the middle of the sensor attachment site in the supine position. To stabilize the body during testing, the players held on to the sides of the plinths while a non-elastic strap was tightly secured across the pelvis. The participant positioning is shown in Figure 2. For each test, participants performed three isometric repetitions, preceded by two familiarization repetitions. They were instructed to apply a standardized pre-tension of 10 N to the brace, which was positioned just proximal to the ankle. From this starting position, following a standardized verbal countdown (“ready, set, go”), participants were instructed to push against the force sensor as quickly and as forcefully as possible with the knee fully extended, aiming to reach maximal force in the shortest possible time. Once maximal force was achieved, participants were instructed to hold the contraction steadily for an additional 5 s. Strong verbal encouragement (e.g., “hold”) was provided throughout the repetition to ensure maximal voluntary effort (15). The rest period between repetitions of each individual test was approximately 30 s, while the rest period between tests targeting different muscle groups was three minutes (24). The preferred side was defined as the push-off leg during a handball jump shot, which is contralateral to the throwing arm. To avoid systematic bias due to fatigue accumulation, the test order was counterbalanced between the preferred and non-preferred sides, as well as across test sequences.

All measurements were recorded at a sampling rate of 1,000 Hz. The signals were automatically processed using the manufacturer's software (ARS for dynamometers, S2P d.o.o, Ljubljana, Slovenija). A moving average filter with a 5 ms window was applied to the force signal. MVIC strength for adduction, abduction, flexion, and extension was determined as the highest force value within a one-second moving average window during the MVICs. The rate of force development was calculated within the first 100 ms of the explosive isometric contraction. Force values were multiplied by leg length and allometrically normalized by a factor of 0.67 to individual body mass, resulting in normalized peak torque (*T*_max_) in Nm/kg^0.67^ and rate of torque development (RTD) in Nm/s/kg^0.67^ (25). *T*_max_ variables demonstrated good to excellent reliability between three test repetitions (ICC_2.k_ = 0.580–0.913), while RTD variables exhibited poor to moderate reliability (ICC_2.k_ = 0.396–0.546). The average of the three *T*_max_ and RTD results (15) were included in the further analysis.

#### 2.2.3 Isometric trunk lateral flexion strength assessment

Trunk lateral flexion strength assessment was conducted using isometric dynamometer (Trunk dynamometer, S2P d.o.o, Ljubljana, Slovenija). Participants stood upright with knees and hips fully extended. One arm was placed across the chest, resting on the opposite shoulder, while the other arm was positioned across the abdomen, resting on the opposite hip. The feet were shoulder-width apart, and the body was held in a neutral, upright posture. Pelvic was firmly fixated in the frontal plane at the iliac crest area and the force transducer pad was positioned the shoulder height contralateral to the pelvic fixation pad (Figure 2, left). Before the initial repetition, the lever arm was measured as a vertical distance between the middle of the force transducer pad and pelvis fixation pad.

Three repetitions (5 s each) of maximal isometric contraction were performed alternately on the preferred and non-preferred sides, preceded by one familiarization repetition. The preferred side was defined as the side of the push-off leg during a handball jump shot, which is contralateral to the throwing arm. Participants were instructed to press maximally against the force transducer pads while stabilizing against the pelvis pads. They were asked to reach maximal voluntary force within approximately 2 s and then hold this maximal effort steadily for an additional 3 s. Strong verbal encouragement (e.g., “hold”) was provided throughout the repetition to ensure maximal voluntary effort. Ankle plantar flexion, knee flexion and hip flexion were not allowed. The presence of the compensatory movements was visually controlled by the researcher. Repetitions with poor test execution were prematurely stopped and repeated. Rest periods between repetitions were 30 s (26).

Measurements were recorded at a sampling rate of 1,000 Hz. The signals were automatically processed using the manufacturer's software (ARS for dynamometers, S2P d.o.o, Ljubljana, Slovenija). A moving average filter with a 5 ms window was applied to the force signal. MVIC strength was determined as the highest force value within a one-second moving average window during the MVICs. Force values were multiplied by trunk lever arm length and allometrically normalized by a factor of 0.67 to individual body mass, resulting in normalized peak torque (*T*_max_) in Nm/kg^0.67^ (25). *T*_max_ variables demonstrated good to excellent reliability between three test repetitions (ICC_2.k_ = 0.910–0.922). The average of the three *T*_max_ results was included in the further analysis.

### 2.3 Statistical analyses

Statistical analyses were performed with SPSS (version 25.0, SPSS Inc., Chicago, USA). Descriptive statistics are reported as mean (standard deviation) and range (minimum–maximum). The normality of the raw data distribution was verified with the Shapiro–Wilk's test. In the first phase of the analysis, intra-session reliability of the SLHJ variables across the three repetitions was calculated using the intraclass correlation coefficient [ICC_2.k_ with 95% confidence intervals (CI)]. According to the latest guidelines, ICC_2.k_ values were interpreted as follows: values <0.50 indicate poor reliability, 0.50 ≤ ICC_2.k_ < 0.75 indicate moderate reliability, 0.75 ≤ ICC_2.k_ < 0.90 indicate good reliability, and ICC_2.k_ ≥ 0.90 indicate excellent reliability (27). Absolute reliability was assessed using the coefficient of variance (CV), with values ≤10% considered acceptable (28). The statistical significance of mean differences among the three repetitions was analysed using a repeated-measures one-way analysis of variance (ANOVA), followed by Tukey's HSD *post hoc* test for pairwise comparisons. In the second phase of the analysis, the strength of linear relationships between hip and trunk strength variables and SLHJ test variables was assessed using Pearson's correlation coefficient (*r*) with Holm's correction applied to statistical significance account for multiple comparisons. The results were interpreted according to recommendations from scientific literature (29) with the following criteria: 0 indicating no correlation, 0.1–0.29 as small, 0.3–0.49 as moderate, 0.5–0.69 as large, 0.7–0.89 as very large, and 0.9–1 as a perfect correlation. Finally, a multiple stepwise regression analysis was performed to identify the hip and trunk strength variables (predictors) that were the strongest predictors of the SLHJ variables (dependent variables). Predictors were included only when their contribution was significant at an alpha level of *p* < .05. Durbin–Watson statistics and collinearity tests were also performed. We set the thresholds for presence of collinearity at five for variance inflation factor. Additionally, a visual inspection of a scatterplot of residuals was done to confirm homoscedasticity of the residuals. For all analyses, the threshold for statistical significance was set at *p* < .05.

## 3 Results

In the Table 1 reliability statistics for modified SLHJ test results for preferred and non-preferred leg are presented. We found poor reliability for preferred leg side deviation (ICC_2.k_ = 0.328) and jump angle (ICC_2.k_ = 0.460) and non-preferred leg jump angle variables (ICC_2.k_ = 0.495). Moderate reliability was found for non-preferred leg side deviation (ICC_2.k_ = 0.698), good reliability for preferred leg jump distance (ICC_2.k_ = 0.865) and excellent reliability was found for non-preferred leg jump distance (ICC_2.k_ = 0.930). According to CV% criteria, only jump distance values were deemed acceptable (CV < 5.7%). ANOVA results revealed statistically significant differences among the three test repetitions only for distance variables (preferred and non-preferred legs; *p* < .01). For the preferred leg, *post hoc* tests revealed significantly greater jump distances in the second and third repetitions compared with the first (both *p* < .01), but no significant difference between the second and third repetitions (*p* = .34). For the non-preferred leg, jump distance increased significantly with each repetition, with all pairwise differences reaching statistical significance (*p* < .05).

**Table 1:** Reliability statistics for modified single-leg horizontal jump for distance.

Side	Variable name	Rep. 1 *M* (*SD*)	Rep. 2 *M* (*SD*)	Rep. 3 *M* (*SD*)	CV (%)	ANOVA		ICC_2.k_ (95% CI)
						*F*	*p*	
Preferred	Distance (m; % leg length)	222.9 (24.2)	228.493 (24.0)	230.0 (27.9)	5.7	8.72	<.01	0.865 (0.823–0.891)
	Deviation (m; % leg length)	2.6 (30.3)	0.7 (19.0)	1.7 (12.6)	120.9	1.01	.37	0.328 (0.087–0.428)
	Jump angle ( °)	0.03 (3.50)	0.25 (5.26)	0.56 (3.27)	130.8	1.03	.36	0.460 (0.232–0.556)
Non-preferred	Distance (m; % leg length)	223.7 (24.2)	231.2 (22.2)	235.1 (21.3)	3.36	34.57	<.01	0.930 (0.882–0.995)
	Deviation (m; % leg length)	7.3 (16.8)	4.9 (9.2)	5.3 (7.9)	159.5	1.38	.26	0.698 (0.591–0.772)
	Jump angle ( °)	1.82 (4.49)	1.11 (2.41)	1.24 (1.94)	193.7	1.69	.19	0.495 (0.268–0.598)

*M*, mean; *SD*, standard deviation; CV, coefficient of variation; CI, confidence interval; *F*, *F*-test statistics; *p*, statistical significance.

Table 2 presents descriptive statistics for hip and lateral trunk strength and SLHJ test results for preferred and non-preferred sides.

**Table 2:** Descriptive statistics for isometric hip strength and modified single-leg jump for distance test results.

Side	Variable type	Variable name	*M* (*SD*)	Minimum	Maximum
Preferred	Hip *T*_max_	Abduction (Nm/kg^0.67^)	8.62 (1.43)	4.81	12.67
		Adduction (Nm/kg^0.67^)	9.74 (2.13)	3.52	14.93
		Flexion (Nm/kg^0.67^)	10.11 (2.17)	2.68	15.17
		Extension (Nm/kg^0.67^)	9.97 (2.18)	3.90	15.65
	Hip RTD	Abduction (Nm/kg^0.67^)	33.57 (17.65)	4.08	11.88
		Adduction (Nm/kg^0.67^)	42.41 (20.6)	4.54	16.50
		Flexion (Nm/kg^0.67^)	61.39 (33.28)	5.40	15.17
		Extension (Nm/kg^0.67^)	39.94 (22.72)	3.95	15.06
	Trunk *T*_max_	Lateral flexion (Nm/kg^0.67^)	16.33 (3.64)	8.63	27.52
	SLHJ	Distance (% leg length)	225.63 (25.93)	112.45	271.60
		Deviation (% leg length)	1.66 (14.22)	−19.87	79.84
		Jump angle (°)	0.23 (2.89)	−5.41	8.84
Non-preferred	Hip *T*_max_	Abduction (Nm/kg^0.67^/s)	8.5 (1.66)	2.73	82.32
		Adduction (Nm/kg^0.67^/s)	10.03 (2.24)	3.64	93.58
		Flexion (Nm/kg^0.67^/s)	10.48 (2.11)	5.84	158.71
		Extension (Nm/kg^0.67^/s)	10.3 (2.28)	6.64	104.52
	Hip RTD	Abduction (Nm/kg^0.67^/s)	39.02 (21.37)	1.48	92.20
		Adduction (Nm/kg^0.67^/s)	47.44 (23.44)	5.63	98.34
		Flexion (Nm/kg^0.67^/s)	68.47 (37.08)	10.28	198.41
		Extension (Nm/kg^0.67^/s)	41.33 (24.63)	1.74	109.88
	Trunk *T*_max_	Lateral flexion (Nm/kg^0.67^)	16.9 (3.78)	8.63	28.98
	SLHJ	Distance (% leg length)	228.62 (23.28)	155.95	276.19
		Deviation (% leg length)	5.63 (8.52)	−22.25	47.83
		Jump angle (°)	1.38 (2.24)	−6.81	12.05

*M*, mean; *SD*, standard deviation; *T*_max_, peak torque during maximal voluntary isometric contractions; RTD, rate of torque development during explosive isometric contractions; SLHJ, single-leg horizontal jump.

Table 3 presents the correlation coefficients with Holm's correction of the statistical significance between hip and trunk strength test results and SLHD test outcomes for both the preferred and non-preferred legs. For the **non-preferred leg**, statistically significant but moderate positive correlations were found between hip extension *T*_max_ and SLHD side deviation (*r* = 0.310, *p* < .05) and between hip flexion RTD and SLHD distance (*r* = 0.308, *p* < .05). No statistically significant correlations were observed between hip and trunk strength and SLDH results for the **preferred leg**.

**Table 3:** Pearson correlation coefficient results between hip and trunk strength test results and modified single-leg horizontal jump for distance test results.

Side	Variable type	Variable name	Single-leg horizontal jump		
			Distance	Deviation	Jump angle
Preferred	Hip *T*_max_	Abduction	0.115 (−0.118; 0.336)	0.033 (−0.195; 0.258)	−0.059 (−0.289; 0.176)
		Adduction	0.171 (−0.062; 0.386)	0.061 (−0.168; 0.284)	−0.062 (−0.292; 0.173)
		Flexion	0.051 (−0.183; 0.28)	0.011 (−0.218; 0.239)	−0.121 (−0.347; 0.117)
		Extension	0.131 (−0.104; 0.352)	0.022 (−0.207; 0.25)	−0.031 (−0.264; 0.205)
	Hip RTD	Abduction	−0.061 (−0.287; 0.172)	0.139 (−0.09; 0.355)	0.176 (−0.06; 0.393)
		Adduction	0.122 (−0.111; 0.343)	−0.032 (−0.257; 0.196)	−0.031 (−0.262; 0.204)
		Flexion	0.047 (−0.187; 0.276)	0.064 (−0.166; 0.289)	0.03 (−0.206; 0.263)
		Extension	−0.082 (−0.308; 0.153)	−0.054 (−0.279; 0.176)	0.11 (−0.128; 0.336)
	Trunk *T*_max_	Lateral flexion	0.083 (−0.147; 0.304)	0.015 (−0.21; 0.238)	−0.024 (−0.253; 0.207)
		Lateral flexion non-preferred	0.095 (−0.135; 0.315)	−0.041 (−0.262; 0.185)	0.048 (−0.184; 0.275)
Non-preferred	Hip *T*_max_	Abduction	0.236 (0.011; 0.438)	0.279 (0.061; 0.472)	0.238 (0.013; 0.44)
		Adduction	0.216 (−0.01; 0.421)	0.245 (0.024; 0.444)	0.206 (−0.02; 0.412)
		Flexion	0.277 (0.054; 0.475)	0.201 (−0.024; 0.407)	0.164 (−0.066; 0.377)
		Extension	0.189 (−0.04; 0.399)	0.310* **(0.092; 0.499)**	0.298 (0.076; 0.492)
	Hip RTD	Abduction	0.163 (−0.065; 0.375)	0.223 (0.003; 0.424)	0.189 (−0.039; 0.397)
		Adduction	0.217 (−0.009; 0.422)	0.261 (0.041; 0.457)	0.274 (0.051; 0.47)
		Flexion	0.308* **(0.088; 0.500)**	0.224 (0.002; 0.427)	0.204 (−0.024; 0.412)
		Extension	0.040 (−0.185; 0.262)	0.237 (0.017; 0.435)	0.208 (−0.017; 0.413)
	Trunk *T*_max_	Lateral flexion preferred	0.087 (−0.138; 0.304)	0.163 (−0.059; 0.369)	0.139 (−0.086; 0.351)
		Lateral flexion	0.037 (−0.187; 0.257)	0.135 (−0.087; 0.345)	0.115 (−0.111; 0.329)

*T*_max_, peak torque during maximal voluntary isometric contraction; RTD, rate of torque development during explosive isometric contraction; SLHJ, single-leg horizontal jump.

Values in bold and marked with * indicate *p* < .05 after a Holm correction.

A multiple regression analysis was conducted to examine predictors of SLHJ performance for the preferred and non-preferred legs. For the **preferred leg** SLHJ distance, adduction *T*_max_ significantly predicted performance (*B* = 3.18, SE = 1.49, *p* = .036), with the regression model explaining 6.3% of the variance [*R* = 0.251, *F*(1, 78) = 4.57, *p* = .036, *R*^2^ = 0.063, adjusted *R*^2^ = 0.051]. Models for preferred leg SLHJ side deviation and angle variables did not include any significant predictors (*p* > .05).

For the **non-preferred leg** SLHJ distance, flexion RTD significantly predicted performance (*B* = 0.20, SE = 0.07, *p* = .006), with the regression model explaining 10.1% of the variance [*R* = 0.318, *F*(1, 78) = 8.01, *p* = .006, *R*^2^ = 0.101, adjusted *R*^2^ = 0.089]. Extension *T*_max_ and abduction *T*_max_ together significantly predicted non-preferred leg SLHJ side deviation (extension *T*_max_: *B* = 0.95, SE = 0.43, *p* = .031; abduction *T*_max_: *B* = 1.12, SE = 0.58, *p* = .054), with the model explaining 14.5% of the variance [*R* = 0.380, *F*(2, 77) = 6.09, *p* = .004, *R*^2^ = 0.145, adjusted *R*^2^ = 0.129]. Extension *T*_max_ alone accounted for 9.4% of the SLHJ side deviation variance (*B* = 1.18, SE = 0.43, *p* = .008). Lastly, the extension *T*_max_ significantly predicted non-preferred leg SLHJ angle (*B* = 0.29, SE = 0.11, *p* = .013), with the regression model explaining 8.4% of the variance [*R* = 0.290, *F*(1, 78) = 6.54, *p* = .013, *R*^2^ = 0.084, adjusted *R*^2^ = 0.073].

## 4 Discussion

This was the first study to investigate side deviation from the expected jump trajectory during the SLHJ test and to examine hip and trunk strength as determinants of SLHJ distance and directional outcomes in elite male team handball players. The analysis of SLHJ measurement characteristics demonstrated good-to-excellent reliability for jump distance but poor-to-moderate reliability for directional variables. Moreover, small-to-moderate correlations were observed between hip and trunk isometric strength and SLHJ performance outcomes, but only for the non-preferred push-off leg. Hip adduction *T*_max_ predicted jump distance for the preferred leg, hip flexion RTD predicted jump distance for the non-preferred leg, and hip extension *T*_max_ predicted side deviation and angle for non-preferred leg jumps, with the regression models explaining 6%–15% of the variance. These findings partially support the hypothesized associations between proximal muscle strength and the control of SLHJ direction, while also highlighting the limitations of static strength assessments in predicting dynamic performance. Furthermore, the present results provide reference values for hip and trunk strength, as well as horizontal jump performance, in elite handball players.

### 4.1 Reliability of the modified single-leg jump for distance test results

While the SLHJ test is well established as a reliable and valid tool for assessing unilateral leg power and functional symmetry (7, 8) the addition of directional variables such as side deviation from the expected jump trajectory and jump angle was intended to capture more complex aspects of neuromuscular control, particularly in the frontal plane. However, the results of our study revealed lower than expected intra-session reliability for these directional variables without major differences between preferred and non-preferred legs. Regardless of the leg, jump distance exhibited good-excellent (ICC_2.k_ = 0.87–0.93) reliability, while side deviation and jump angle (ICC_2.k_ = 0.33–0.70) demonstrated moderate to poor reliability. These findings emphasize the need for cautious interpretation of side deviation from the expected jump trajectory outcomes, despite their potential biomechanical relevance. This is consistent with earlier evidence suggesting that directional control during dynamic tasks is more variable and influenced by multiple interacting factors including motor coordination, balance, and attentional strategies (16, 20, 30). As such, although side deviation from the expected jump trajectory may provide additional qualitative insight into movement strategy, it cannot yet be considered a reliable outcome measure for individual monitoring without further standardization. One possible explanation for the lower reliability of the jump angle variables is that small differences in jump distance and side deviation values between repetitions (i.e., the numerator and denominator when calculating the angle) can result in greater relative uncertainty in the ratio, yielding a metric that may reflect a different construct. These findings are consistent with previous research indicating that ratio-based metrics often exhibit lower reliability than their constituent measures because variability from both components is combined, increasing measurement error (31). We should also note that jump distance increased significantly with the number of jump repetitions, whereas this was not the case for the directional measures of the SLHJ. This increase may reflect familiarization or potentiation effects across repetitions (32). Averaging three repetitions, as performed in our study, is recommended to further reduce the noise-to-signal ratio (33, 34), that is, to reduce the random error associated with a single repetition and thereby improve the credibility of the results. As the majority of jumps in handball are performed using a single leg, typically contralateral to the throwing arm, it could be speculated that this preferred push-off leg would demonstrate higher reliability (i.e., more consistent performance) across repeated jump repetitions. However, this was not confirmed in the present study, as only minor differences in reliability parameters were observed between the preferred and non-preferred legs. These findings indicate that the same testing protocol can be reliably applied to assess SLHJ performance in both legs of elite handball players, for example, allowing coaches and sports scientists to evaluate SLHJ performance in a single session without requiring extra familiarization for the non-preferred leg.

### 4.2 Associations between modified single-leg jump for distance results, hip and lateral trunk strength

Although hip and trunk muscles are known to contribute significantly to jump distance (5) and to pelvic stability and directional control during unilateral movements (17, 18), the correlations observed in our study were lower than expected. Specifically, no significant correlations were found for the preferred leg between SLHJ and strength variables. And multiple regression analysis revealed that only adduction *T*_max_ was a significant predictor of preferred leg jump distance, explaining 6.3% of the variance. This suggests that factors other than hip and trunk strength, such as inter-muscular coordination (35), may contribute to jump performance more than strength variables.

The contribution of maximal hip adductor strength to the **preferred leg** SLHJ distance is an interesting finding, to the authors' knowledge not previously reported for a single horizontal jump performed on one leg. Maximal hip adductor strength likely contributes both to horizontal (propulsive) force generation through hip extension and to pelvic stability in the frontal plane during unilateral push-off. The adductor magnus functions as a hip extensor when the hip is flexed (36), a position resembling the semi-squat posture observed during the transition from the eccentric to concentric phase of the SLHJ. In this context, it assists the gluteus maximus and hamstrings in producing hip extension force (37), thereby enhancing horizontal propulsion during the take-off phase. During unilateral tasks, the adductors also play a key role in stabilizing the pelvis and maintaining femoral alignment together with the hip abductors (38). This coordination counteracts excessive hip abduction range of motion that could impair kinetic-chain energy transfer between joints during push-off action. Our results are similar to those of Unal-Suzer et al. (38), however, their findings relate to a consecutive single-leg triple-hop distance rather than the SLHJ distance examined in our study. In their study, maximal hip adductor strength explained 50% of the variance in jump distance, considerably more than in the present study. This may emphasize the greater contribution of maximal hip adductor strength to jump performance during more intensive eccentric–concentric actions, likely due to its role in pelvic stabilization and the generation of higher muscle forces during consecutive single-leg landings and push-offs. Furthermore, adequate adductor strength supports dynamic balance and intersegmental coordination, both of which are essential for maintaining jump trajectory control and effective landing mechanics. This pattern suggests that muscles responsible for frontal-plane control may play a compensatory role when neuromuscular control is challenged, consistent with previous reports highlighting the biomechanical importance of proximal muscle groups in segmental alignment and energy transfer (5). It may also be relevant that adductor *T*_max_ in our study exhibited greater variability among participants (CV = 22%) compared with, for example, abduction *T*_max_ (CV = 17%), potentially making it a more sensitive indicator of strength deficits and, consequently, SLHJ performance.

For the **non-preferred leg**, one significant correlation was observed for jump distance and one for side deviation. Multiple regression analyses supported correlation analysis findings, showing that flexion RTD predicted non-preferred-leg jump distance (explaining 10% of the variance), whereas extension *T*_max_ and abduction *T*_max_ together predicted side deviation (14% of variance explained), and extension *T*_max_ alone predicted jump angle (8.4% of variance explained). These results indicate that hip muscle strength is a stronger determinant of single-leg jump performance for the non-preferred leg compared with the preferred push-off leg. Another noteworthy finding is the correlation between hip flexion RTD and jump distance. While hip flexors are not primary contributors to propulsive momentum during jumping, it may be speculated that some participants adopted a technique involving a forceful upward pull of the knee toward the trunk after take-off, generating additional momentum. This could represent a compensatory strategy for suboptimal jump technique or comparatively lower strength of the knee and ankle extensors in the non-preferred push-of leg. Although the explained variance was smaller, these findings partially align with those of Unal-Suzer et al. (38), who found hip flexion RFD to be a significant determinant of triple-hop mechanical work (37% of variance explained). This finding may also be related to adaptations from the frequent use of the handball approach jump shot technique, in which the non–push-off leg is explosively flexed at the hip during take-off. Over time, this repeated movement may lead to increased explosive and maximal strength of the non-preferred leg hip flexors in handball players. Consequently, when performing the SLHJ in our study, players may have leveraged these adaptations to enhance jump distance.

Furthermore, participants with stronger hip extensors and abductors tended to jump more medially than the expected trajectory using non-preferred leg. This finding may be explained by the combined action of the gluteus maximus (the primary hip extensor) and gluteus medius (the primary hip abductor) (37). When these muscles contract simultaneously during single-leg push-off, their resultant force vector acts diagonally, directed backward and outward relative to the pelvis. Because the foot is fixed on the ground, the equal and opposite ground reaction force is directed forward and slightly inward (medially), propelling the body toward the midline. Consequently, stronger individuals in these muscle groups may generate greater diagonal push-off forces, producing a more medial jump trajectory. This pattern likely reflects a hip-dominant propulsion strategy among these participants, where enhanced extensor and abductor strength contributes to stability and force production at the expense of a perfectly straight trajectory.

The absence of associations between lateral trunk flexor strength and SLHJ variables likely reflects the stabilizing, rather than propulsive, role of these muscles during the jump. Lateral trunk flexors help maintain segmental alignment and efficient force transfer without directly contributing to propulsion. Moreover, the static nature of the isometric trunk test likely failed to capture the dynamic eccentric–concentric actions required in ballistic movements (20, 21). This aligns with prior evidence indicating that isolated isometric strength measures are weak predictors of dynamic jump performance (39, 40). Importantly, intervention studies have shown that improving hip muscle function can meaningfully alter landing strategies and reduce frontal-plane loading (6), underscoring the functional importance of these muscles despite the limited predictive value of static strength measures for single-leg jump distance in our study. Finally, the use of isometric testing conditions may partly explain the small-to-moderate correlations observed, as such assessments do not capture the concentric and eccentric dynamics, timing, and intermuscular coordination required during ballistic tasks like the SLHJ (5, 12). Interestingly, despite years of sport-specific handball training that predominantly involves single-leg jumping, hip strength was a weaker predictor of jump performance for preferred push-off leg. It could be argued that continuous unilateral jumping during handball training would enhance hip and trunk strength and be reflected in superior single-leg jump performance. However, this does not appear to be the case. Instead, other factors, such as jump coordination, may have a greater influence on performance of the preferred leg. Conversely, jump distance on the non-preferred leg may reflect hip strength more strongly, likely due to less optimal intramuscular coordination or jump technique. These findings indicate that, in practice, both legs should be assessed. Sport-specific asymmetry therefore exists and should be considered; however, optimal thresholds for reducing injury risk or improving bilateral performance (41) remain to be established for SLHJ test and should be examined in future research.

### 4.3 Limitations and future perspectives

Several limitations should be acknowledged when interpreting the present findings. First, the modified SLHJ protocol allowed unrestricted use of the arms during take-off and landing, which increased the handball-specific execution of the test and made it more comparable to the actual movement demands in team handball. However, this may also have introduced greater variability and contributed to the poor reliability of directional variables. Future studies could therefore consider a standardized, hands-free version to better isolate lower-limb and trunk control. Due to the greater variability of the results, we suggest that future research examining directional outcomes should include more than three repetitions to further reduce the noise-to-signal ratio and improve the credibility of the results (33, 34). Nevertheless, the optimal number of repetitions remains to be determined and should be explored in future studies. Second, the use of isometric strength testing may not fully capture the dynamic nature of force production and control during jumping. Incorporating more dynamic and sport-specific strength assessments could improve predictive value. Additionally, the hip RTD results showed lower reliability (greater variability between repetitions) compared with *T*_max_ results, which may have made it harder to detect true relationships with jump performance, despite averaging multiple repetitions. Furthermore, the SLHJ was performed from a standing position, which may not accurately reflect typical handball game situations (e.g., approach jumps). Regardless of the observed differences in strength predictors between limbs (where only one predictor was identified for the preferred leg) it can be speculated that this test may not be sufficiently specific to capture handball-specific differences in strength adaptations between limbs. Third, although statistically significant, the predictive values of the measured strength variables to jump variables were relatively low (up to 14% of the variance), suggesting that jump distance and side deviations from the expected jump trajectory are influenced by multiple factors beyond isolated muscle strength. Lastly, while the sample consisted of elite male team handball players, generalizability to other sports, female athletes, or sub-elite populations remains uncertain. Given the playing positional demands in team handball, future research may also consider analyzing differences in jump mechanics and control between playing positions, especially since previous studies have shown clear anthropometric and physical distinctions across positions (1, 4).

Despite the observed limitations, the findings of this study suggest that the modified SLHJ test, which incorporates both traditional jump distance and novel side-deviation variables, may provide valuable insights into jump directional control during unilateral tasks. While the directional variables demonstrated lower reliability, their potential to reflect neuromuscular deficits and compensatory strategies warrants further investigation. The multiple small-to-moderate associations observed between hip and trunk strength and SLHJ outcomes highlight the importance of a multifactorial approach when evaluating performance and injury risk, with separate consideration of the preferred and non-preferred jump legs. The modified SLHJ may serve as a practical, field-based screening tool for identifying jump-related compensatory strategies associated with hip strength, particularly in the non-dominant leg, in handball players. Finally, it should be emphasized that the present findings are based on a cross-sectional design rather than a longitudinal one. To confirm whether improvements in hip strength influence modified SLHJ outcomes for preferred and non-preferred legs, interventional studies are required. This test represents an initial step toward a simple, field-based assessment of jump performance that simultaneously reflects hip strength, particularly in the non-dominant leg. With improved standardization and further validation, especially of the directional variables, the test could evolve into a more comprehensive tool for performance profiling and injury prevention strategies in elite team sports.

### 4.4 Practical applications and conclusion

The modified SLHJ side-deviation and jump-angle measures in the non-preferred leg may help identify hip strength–related factors that are not captured by jump distance alone. However, due to their lower reliability, coaches and clinicians working with handball players should interpret SLHJ directional outcomes with caution, particularly when using them to monitor progress or inform return-to-play decisions. In addition to jump distance, averaging more than three repetitions appears necessary to obtain reliable results, regardless of the tested leg; however, future studies are required to confirm this recommendation. Hip adduction *T*_max_ and hip flexion RTD were predictors of jump distance for the preferred and non-preferred legs, respectively. Furthermore, hip extension *T*_max_ predicted a more medial jump trajectory in the non-preferred leg. These findings suggest that handball players' push-off leg preference influences SLHJ outcomes; therefore, both legs should be assessed and interpreted accordingly.

The current version of the SLHJ may, in handball practice, serve as a field-based general movement screening test. Its use should be combined with other functional assessments, such as evaluations of landing mechanics or trunk control. The modified SLHJ may help identify specific hip strength–related factors that are not captured by jump distance alone. However, any conclusions regarding hip strength or strength deficits derived from jump distance and directional measures, including deviations from the reference values presented in this study, should be confirmed using laboratory-based strength testing methods before drawing definitive conclusions or implementing training or rehabilitation interventions.

## Data Availability

The raw data supporting the conclusions of this article will be made available by the authors, without undue reservation.
